# A new immunodeficient Duchenne muscular dystrophy rat model to evaluate engraftment after human cell transplantation

**DOI:** 10.3389/fphys.2023.1094359

**Published:** 2023-04-10

**Authors:** Masae Sato, Megumi Goto, Keitaro Yamanouchi, Hidetoshi Sakurai

**Affiliations:** ^1^ Department of Clinical Application, Center for iPS Cell Research and Application (CiRA), Kyoto University, Kyoto, Japan; ^2^ Department of Veterinary Physiology, Graduate School of Agricultural and Life Sciences, The University of Tokyo, Tokyo, Japan

**Keywords:** DMD, rat model, immunodeficient, cell transplantation, pathology

## Abstract

Duchenne muscular dystrophy (DMD) is an X-linked fatal muscular disease, affecting one in 3,500 live male births worldwide. Currently, there is no cure for this disease, except for steroid-based treatment to attenuate disease progression. Cell transplantation therapy is a promising therapeutic approach, however, there is a lack of appropriate animal models to conduct large-scale preclinical studies using human cells, including biochemical and functional tests. Here, we established an immunodeficient DMD rat model and performed exhaustive pathological analysis and transplantation efficiency evaluation to assess its suitability to study DMD. Our DMD rat model exhibited histopathological characteristics similar to those observed in human patients with DMD. Human myoblasts demonstrated successful engraftment following transplantation into these rats. Therefore, this immunodeficient DMD rat model would be useful in preclinical studies to develop cellular transplantation therapies for DMD.

## 1 Introduction

Duchenne muscular dystrophy (DMD) is caused by multiple mutations in the *DMD* gene, which is located on the X chromosome (Hoffman et al., 1987). The *DMD* gene encodes dystrophin, which connects the muscle cell cytoskeleton to the extracellular matrix (Robert, 2001). DMD is an intractable and fatal muscle disease that causes chronic inflammation and muscle degradation. Patients with DMD are usually diagnosed at 3–5 years of age and lose the ability to walk at approximately 12 years of age. Subsequently, they require respiratory support and suffer from cardiomyopathy in their early teens (Fairclough et al., 2013). Currently, there is no curative treatment for DMD, however, dystrophin restoration is being investigated as a possible treatment option, and several therapeutic approaches have been investigated, including exon-skipping, gene therapy by oligonucleotides (Fairclough et al., 2013) (Beytía et al., 2012), and cell transplantation (Sun et al., 2020). Preclinical trials in dogs and mice have recently been reported using these approaches (Beytía et al., 2012) (Nowak and Davies, 2004). However, milder phenotypes were reported in C57BL/10-mdx mice than in humans (Nakamura and Takeda, 2011), while dogs showed greater individual variation. Proof-of-concept studies using DMD model animals are necessary to develop novel therapies that restore dystrophin expression in DMD. Thus, we used a rat model with fewer genetic differences, and that was more symptomatic of DMD than C57BL/10-mdx mice (Larcher et al., 2014) (Nakamura et al., 2014) (Taglietti et al., 2022). Cell therapy is a promising therapeutic approach for DMD because adult muscle stem cells (MuSCs), or satellite cells (Marg et al., 2014) (Cerletti et al., 2008) (Sacco et al., 2008) (Tanaka et al., 2009) (Xu et al., 2015) (Montarras et al., 2005), retain robust regeneration potential for injured muscle cells. Recently, it was reported that skeletal MuSCs induced from induced pluripotent stem (iPS) cells can be grafted into mice, and we observed that muscle tone was restored after transplantation (Zhao et al., 2020). Preclinical studies of cell transplantation therapies involve transplanting human cells into animals, requiring immunosuppressive agents to prevent rejection of the transplanted human cells. Additionally, the prolonged use of immunosuppressive drugs is discouraged as it could modify the disease state (Samata et al., 2015). Nude rats with T cell dysfunction are capable of successful human cell engraftment and have been used as immunodeficient animal models (Hanes, 2006). Nude rats are also used in preclinical studies of induced pluripotent stem cell-derived dopaminergic progenitor cells for the treatment of Parkinson’s disease (Doi et al., 2020) Therefore, future research regarding clinical applications and basic analysis of DMD should be conducted using an immunodeficient DMD rat model.

In this study, we generated an immunodeficient DMD rat model capable of human cell engraftment by crossing a DMD model rat which shows no revertant fiber in skeletal muscle (Nakamura et al., 2014) with a nude rat. We conducted histopathological evaluation of the novel rat model to confirm successful engraftment after immortalized human myoblast cell transplantation.

## 2 Materials and methods

### 2.1 Animals

All experiments involving the use of animals were performed in accordance with the Guide for the Care and Use of Laboratory Animals of the University of Kyoto, and the procedures were approved by the Institutional Animal Care and Use Committee of the University of Kyoto (approval number 19-119). Animals were maintained at 23°C with a 12/12 h light/dark cycle, with lights on at 08:00 a.m. and were fed *ad libitum*.

### 2.2 Generation of immunodeficient DMD rat model

We used the Wistar–Imamichi strain DMD rat model (DMD rat model: X^Dmd^Y) carrying mutations in exons 3 and 16 of the *Dmd* gene (Nakamura et al., 2014). Immunodeficient F344/NJcl.Cg-Foxn1^rnu^ rats (nude rat: Foxn1^rnu/rnu^) were purchased (CLEA Japan, Inc., Tokyo, Japan). Immunodeficient DMD rats were established by crossing a Foxn1^rnu/rnu^ male rat with an X^Dmd^X female rat. X^Dmd^ was identified by genotyping the litter population (Nakamura et al., 2014). The following rat genotypes were used in this study: X^Dmd^Y Foxn1^rnu/rnu^ (DMD nude rats), XX Foxn1^rnu/rnu^ or XY Foxn1^rnu/rnu^ rats (WT nude rats) and X^Dmd^Y Foxn1^rnu/+^(immunocompetent DMD rat model).

### 2.3 Creatine kinase activity

Creatine kinase activity in the serum of DMD nude rats and their age-matched WT nude rats (1-, 5-, and 12-months-old) was measured using a Fuji Dri-Chem system by Oriental Yeast Co., Ltd. (Tokyo, Japan).

### 2.4 Urinary titin and creatine measurement

Urine samples were obtained from 1-, 5-, and 12-month-old WT nude and DMD nude rats. Urinary titin was measured using a Mouse Titin N-Fragment Assay Kit (27,602, Immuno-Biological Laboratories Co., Ltd.). Urinary creatinine (Cr) concentrations were measured using a Lab Assay Creatinine kit (636–51011, Fujifilm Wako Pure Chemical Corporation Ltd.) (Maruyama et al., 2016).

### 2.5 Grip test

Forelimb strength was determined by a muscle relaxation measurement device (no. BS-TM-RM, Brain Science idea, Osaka, Japan). The study was conducted on 5 to 6 and 9-month-old WT nude and DMD nude rats. Tails of rats gripping a wire mesh plate, which was equipped with a devise, were pulled horizontally and the maximum tension was recorded. In 3 serial times of pulling, the average forelimb muscle tension was used as the value for muscle strength. All trials were performed by one examiner.

### 2.6 Open-field test

The open-field test was used to assess vertical (rearing) activities. The rats were placed in a cylinder and their behavior was recorded using a camera for 3 min to count the number of rearing behaviors. The study was conducted on 12-month-old WT nude and DMD nude rats. All experiments were conducted at the same location (2 m above the floor) and under constant illumination.

### 2.7 Hu5/KD3 cell culture

Human immortalized myoblast cell line Hu5/KD3 was provided by the National Center for Geriatrics and Gerontology (Obu, Aichi, Japan) (Shiomi et al., 2011). The cells were maintained on collagen type I-coated dishes (4010-010, Iwaki Glass Co., Ltd.), in high glucose Dulbecco’s modified Eagle’s medium (DMEM; 08,488-55, Nacalai Tesque) supplemented with 20% fetal bovine serum (FBS; 556-33865, Gibco, ThermoFisher Scientific), 2% Ultroser G (15,950-017, Biosepta, Pall Life Sciences), 1% L-glutamine (15,950-017, Invitrogen), and 0.5% penicillin streptomycin mixed solution (09,367-34, Nacalai Tesque).

### 2.8 Preparation of polymer solution

Gelatin (Kindly provided from Dr. Yasuhiko Tabata, Kyoto University) and hyaluronic acid (Kindly provided from Dr. Yasuhiko Tabata, Kyoto University) were dissolved in 20% FBS DMEM without Ultroser G, at 1% w/v at 37°C in a water bath. A mixed polymer solution was prepared by mixing the hyaluronic acid solution with gelatin solution in a 1:4 volume ratio (Miura et al., 2022).

### 2.9 Transplantation studies

4-week-old WT and DMD nude rats were anesthetized with 2% isoflurane (704239096, VIATRIS) and cryoinjury was performed to model muscle injury. The tibialis anterior (TA) was exposed, and cryoinjury was performed on both TA muscles three times for 12 s each by direct contact of the TA muscles with a cryoprobe dipped in liquid nitrogen (Nalbandian et al., 2021). Following cryoinjury, 6 × 10^6^ Hu5/KD3 cells were mixed with the polymer solution, as described above, and injected into the left and right TA muscles. The rats were sacrificed either 3 weeks or 3 months after transplantation, and the TA muscle samples were collected and analyzed by immunohistochemical staining.

### 2.10 Histological analyses of skeletal muscle tissues

Histological analyses were performed as previously described (Takenaka-Ninagawa et al., 2021). The skeletal muscle tissues were mounted in tragacanth gum (206–02242, Fujifilm Wako Pure Chemical Corporation Ltd.) and frozen using liquid nitrogen-cooled isopentane. Paraffin-embedded skeletal muscle tissues were used for hematoxylin & eosin (H&E) staining. Additionally, the heart and the diaphragm were also stained with H&E and Sirius red (SR), which was performed by Applied Medical Research, Inc. (Osaka, Japan). Cryosections (12 µm) of TA muscles were obtained using a cryostat (Leica Biosystems). For samples used for immunostaining, the sections were fixed with 4% paraformaldehyde (PFA; 09154-85, Nacalai Tesque) in phosphate-buffered saline (PBS; 11,482-15, Nacalai Tesque) at 24°C for 20 min. For quantitative analysis of the myofiber minimal Feret’s diameter, cryosections were fixed with methanol at −20°C for 7 min. After fixation, sections were blocked with Blocking one (03,953-95, Nacalai Tesque) for 1 h, followed by incubation with primary antibodies (Supplementary Table S1) diluted in Can Get Signal B (NKB-601X4, Toyobo) overnight at 4°C. Sections were then washed twice with 0.2% Triton X in PBS and incubated with secondary antibodies (Supplementary Table S1) diluted in Can Get Signal B for 1 h. Subsequently, the sections were washed twice with 0.2% Triton X in PBS and the immunostained sections were mounted using Vectashield mounting medium with DAPI (H-1200, Vector Laboratories). Immunofluorescence images were acquired using a Zeiss LSM 700 laser scanning confocal microscope (Carl Zeiss). For quantitative analysis of myofiber minimal Feret’s diameters, 10 fields were randomly selected from sections stained with anti-Pan laminin antibody. The minimal Feret’s diameter and SR-stained positive area per section were calculated using Keyence image analyzer (Keyence Corporation, Osaka, Japan)

### 2.11 Histological analysis of transverse sections

For transverse sections, WT and DMD nude rats were reflux-fixed using 10% formalin (37,152-51, Nacalai Tesque) and then decalcified and paraffin-embedded by Applied Medical Research, Inc. Both H&E and SR staining were performed by Applied Medical Research, Inc. Images were captured using a BZ-X700 microscope (Keyence, Osaka, Japan).

### 2.12 Statistical analysis

All statistical analyses were performed using GraphPad Prism version 9.2.0 for Mac OS (GraphPad Software). Data are expressed as the mean ± SEM. Differences between the groups were analyzed using the two-tailed paired Student’s t*-*test. A *p-*value <0.05 was considered statistically significant. GraphPad Prism was used to calculate survival curves (Kaplan-Meier) with Mantel-Cox test and to construct graphs for each group. All live WT nude rats were discontinued for follow-up at 76 weeks.

## 3 Results

### 3.1 Physical, behavioral, and biochemical characteristics of immunodeficient DMD rats

**FIGURE 1:**
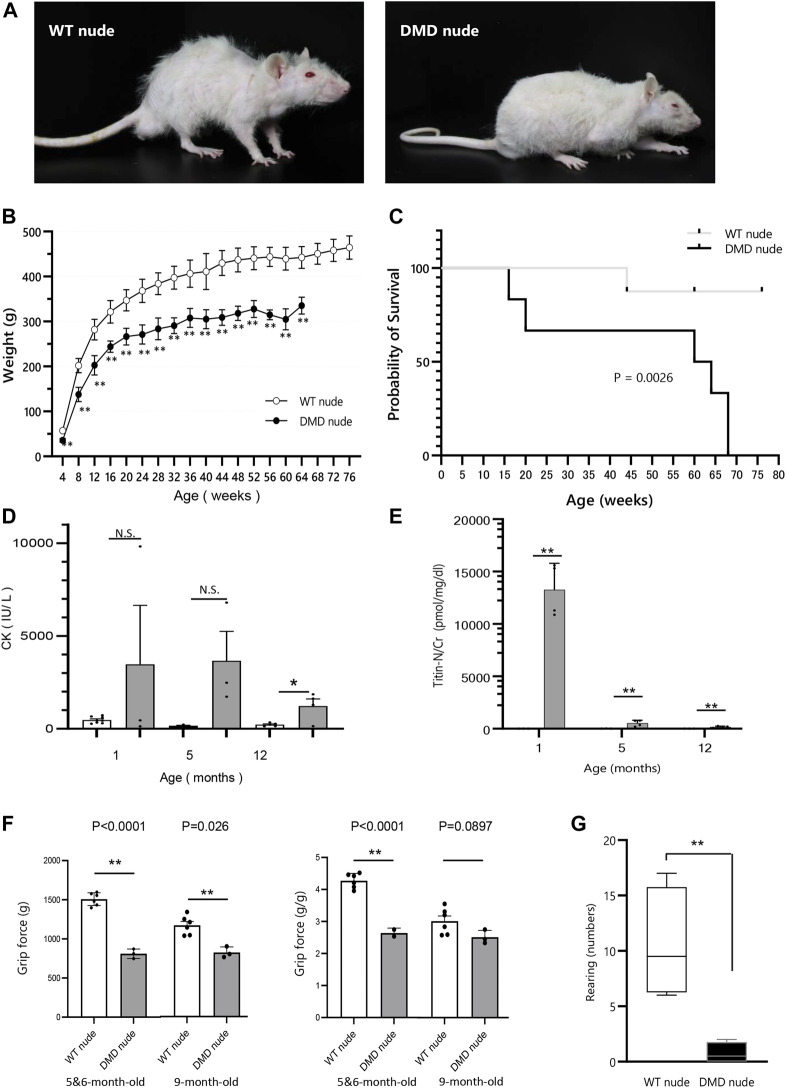
Generation of immunodeficient Duchenne muscular dystrophy (DMD) rat model. **(A)** Representative images of 12-month-old wild type (WT) and DMD nude rats. **(B)** Body weight comparison of WT and DMD nude rats. (WT nude rat: *n* = 8, DMD nude rat: *n* = 7). **(C)** Kaplan-Meier curves comparing survival rates of WT and DMD nude rats. Mantel-Cox test, *p* = 0.0026. (WT nude rat: *n* = 8, DMD nude rat: *n* = 7). **(D)** The serum creatine kinase (CK) activity in WT and DMD nude rats (WT nude rats: *n* = 8, 3, and 5; DMD nude rat: *n* = 3, 3, and 3). **(E)** The urinary titin concentrations in WT and DMD nude rats (WT nude rats: *n* = 3 for all groups; DMD nude rat: *n* = 4 for all groups). **(F)** Quantification of maximum muscle strength by grip test. The study was conducted on 5 to 6 and 9-month-old WT nude and DMD nude rats. (WT nude rat: *n* = 2, 2, DMD nude rat: *n* = 1, 1). **(G)** Motor behavior was examined using an open-field test. WT and DMD nude rats at 12-month-old were individually placed in the cylinder for 3 min (WT nude rat: *n* = 4, DMD nude rat: *n* = 4). *p < 0.05, **p < 0.01.

F344/NJcl.Cg-Foxn1^rnu^ rats are immunodeficient nude rats with defective T cell function (Hanes, 2006). We crossed Wistar–Imamichi strain DMD model rats (Nakamura et al., 2014) with F344/NJcl.Cg-Foxn1^rnu^ rats (WT nude rat) to establish an immunodeficient DMD rat model (DMD nude rat). The body size of 12-month-old DMD nude rats was smaller than that of the WT nude rats (Figure 1A). DMD nude rats often remained close to the floor due to decreased muscle strength in their extremities (Figure 1A) and weighed less than WT nude rats starting at 4-week-old (Figure 1B). The survival curves for WT and DMD nude rats revealed that half of the DMD nude rats died at 60-week-old, with no surviving animals beyond 68-week-old. The life span of DMD nude rats was significantly shorter than that of WT nude rats, as seen from the survival curves (Figure 1C). Human patients with DMD display muscle damage characterized by increased CK activity in the serum and increased titin levels in urea (Goto et al., 1967) (Maruyama et al., 2016). The CK activity level in 1-month-old DMD nude rats was significantly increased compared to that in WT nude rats, which decreased after 12-month-old (Figure 1D). A similar trend was observed for urinary titin (Figure 1E). Furthermore, to measure the muscle function impairments of DMD nude rats quantitatively, grip tests were performed on 5 to 6 and 9-month-old WT nude rats and DMD nude rats. Analysis of grip force showed significant reduction of muscle function in DMD nude rats compared to WT nude rats at both 5 to 6-month-old and 9-month-old (Figure 1F, left). Considering the low body weight in DMD nude rats, a statistically significant reduction of approximately 36% in grip force normalized by body weight was also observed in DMD nude rats (approximately 2.74 g/g) compared to WT nude rats (approximately 4.27 g/g) at 5 to 6-month-old (Figure 1F, right). Spontaneous locomotion measurement is an important analysis to confirm the phenotype in DMD animal models (Larcher et al., 2014). Thus, we measured a horizontal activity such as rearing and observed a significant reduction of 93% ± 0.1% in DMD nude rats compared to that in WT nude rats (Figure 1G). These results indicate that DMD nude rats display progressive muscle tissue damage followed by severe muscle weakness. In addition, significant weight loss was observed in DMD rats, with a shorter life span compared to WT nude rats.

### 3.2 Pathological analysis of the skeletal muscle tissues in DMD nude rats

**FIGURE 2:**
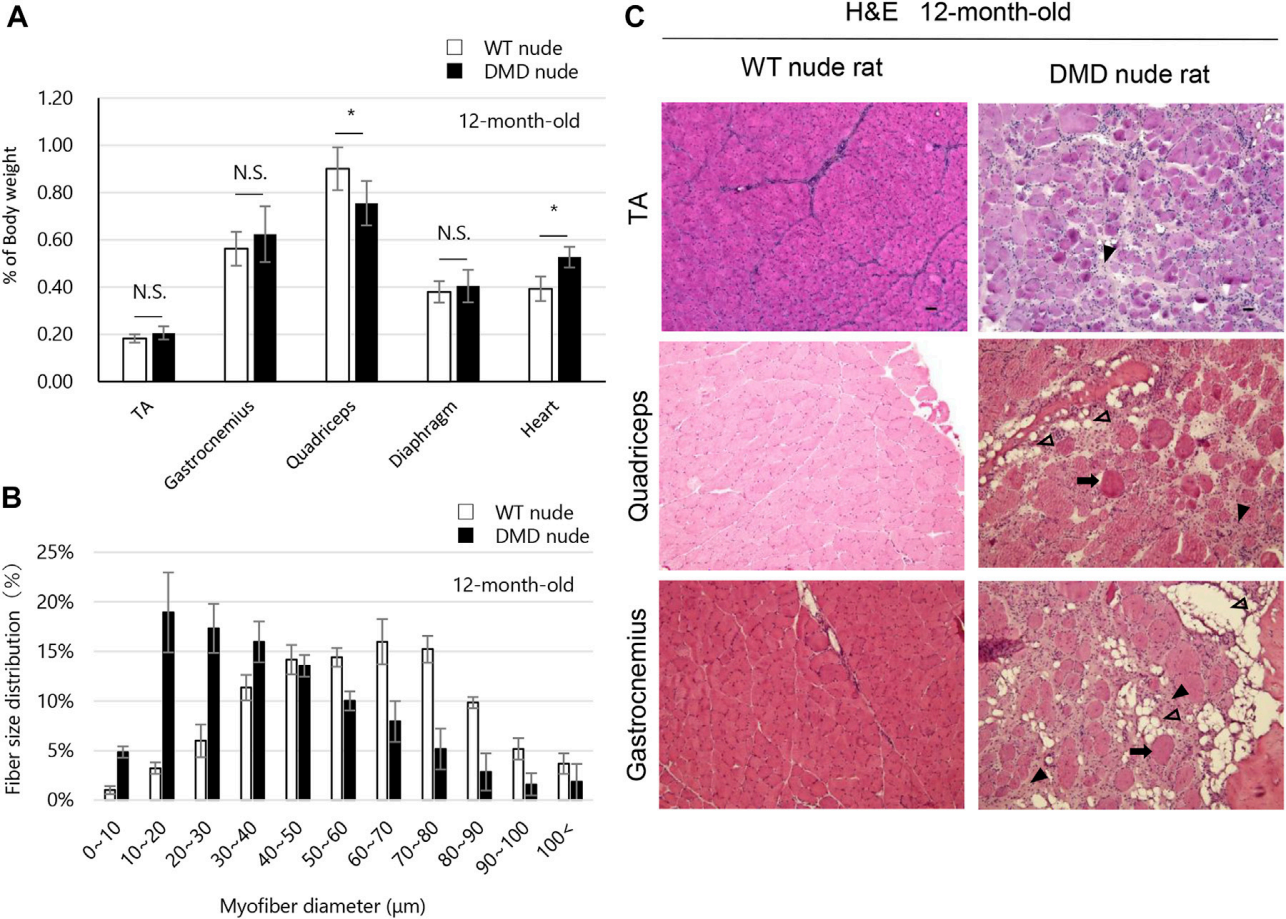
Phenotypic analysis of the skeletal muscles of DMD nude rats. **(A)** The percentage of body weight represented by each muscle tissue at the indicated ages in WT and DMD nude rats. N.S. non-significant, *p < 0.05. **(B)** Histological changes in the fiber diameter in 12-month-old WT and DMD nude rats. **(C)** Representative images of hematoxylin & eosin (H&E) staining of the tibialis anterior (TA), quadriceps, and gastrocnemius muscle sections in 12-month-old WT and DMD nude rats. The symbols, ➡, ▼, and ▽, indicate necrotic myofibers, fibrosis, and adipogenesis, respectively. Scale bar = 50 µm.

The weights of each skeletal muscle tissue, the diaphragm, and the heart of WT nude and DMD nude rats were compared at 1, 5, and over 12-month-old (Table 1). We observed decreased skeletal muscle weights in DMD nude rats compared to that in WT nude rats, reflecting the small body size of DMD nude rats, except for the weight of the TA muscle at 5 months (Table 1). Although no statistically significant difference was observed, the increased TA muscle weight in DMD nude rats indicated a tendency towards pseudohypertrophy. Due to body weight differences between WT and DMD nude rats (Figure 1B), we normalized the weights of various skeletal muscles, such as the diaphragm and the heart, to their respective body weights (% of body weight for each tissue). Among skeletal muscles, the percentage of body weight for the quadriceps muscle was significantly lower in 12-month-old DMD nude rats than in WT nude rats (Figure 2A), indicating that the proximal muscle atrophied more than the distal muscle. However, no significant differences were observed between the two groups in the percentage of body weight for the muscles at 1 and 5-month-old (Supplementary Figure S1). Furthermore, quantification of histological changes in the fiber diameter on the quadriceps muscle revealed an overall decreased fiber size in 12-month-old DMD nude rats compared to that in WT nude rats (Figure 2B). Moreover, histopathological evaluation of the TA, quadriceps, and gastrocnemius muscles using H&E staining revealed necrotic fibers and inflammatory cell infiltration in 12-month-old DMD nude rats compared to that in age-matched WT nude rats (Figure 2C), and increased adipogenesis in the quadriceps and gastrocnemius muscles in DMD nude rats (Figure 2C). We also observed significantly increased percentage of body weight of the heart in 12-month-old DMD nude rats compared to that in WT nude rats (Figure 2A). Thus, DMD nude rats displayed cardiac hypertrophy and pathological heart dysfunction at 12-month-old.

**TABLE 1:** Total body weight and weights of various skeletal muscles in WT and DMD nude rats at 1, 5, and over 12-month-old.

Age	1month-old		5 month-old		Over 12 months old	
	WT	DMD	WT	DMD	WT	DMD
Muscle weight (mg)						
Tibialis anterior muscle	324.4 ± 26.6	217.8* ±13.0	629.7 ± 20.3	763.4 ± 60.6	734.7 ± 15.3	700.9 ± 51.7
Gastrocnemius	863.1 ± 57.8	804.2 ± 5.0	2034.1 ± 39.3	2058.0 ± 171.4	2338.4 ± 104.3	2114.9 ± 196.6
Quadriceps femoris	1284.3 ± 82.5	1254.6 ± 7.0	3166.3 ± 84.4	2702.6 ± 107.2	3734.8 ± 96.3	2643.8** ±171.9
Diaphragm	601.0 ± 47.8	698.4 ± 79.0	1281.9 ± 67.3	1375.4 ± 42.2	1587.3 ± 91.1	1396.3 ± 112.4
Heart	743.8 ± 70.4	773.4 ± 25.4	1328.6 ± 41.4	1327.6 ± 70.0	1630.5 ± 73.4	1807.3 ± 82.1
Number of samples	n = 4	n = 3	n = 3	n = 5	n = 8	n = 6

Data show the means and ±SEM., The number of samples is 4 for the Tibialis anterior muscle only over 12-month-old WT nude rat. *p < 0.05; **p < 0.01.

### 3.3 Pathological analysis of the heart in DMD nude rats

**FIGURE 3:**
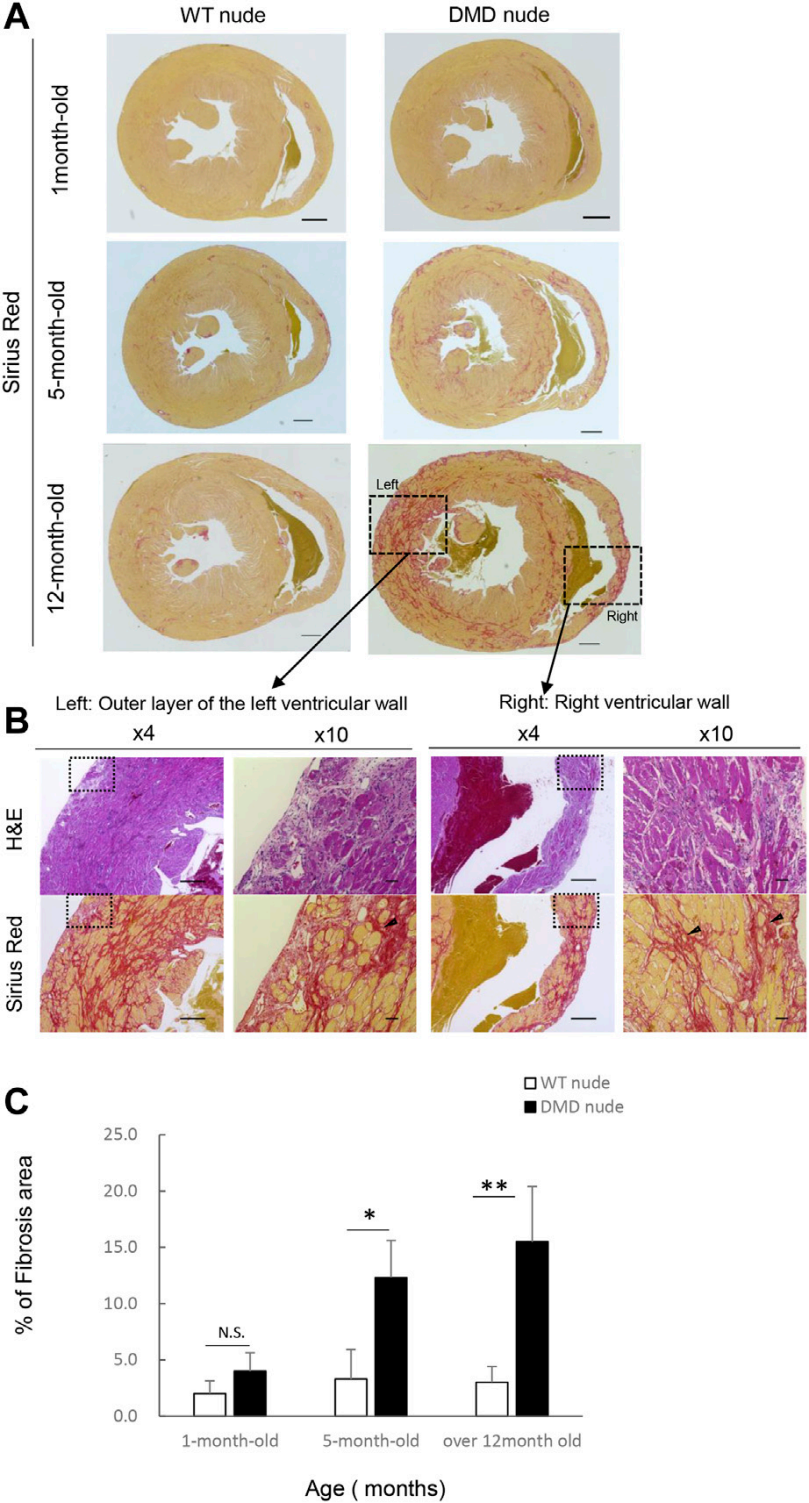
Severe and progressive muscle changes in the heart of DMD nude rats. **(A)** Representative images of Sirius red (SR)-stained hearts of WT and DMD nude rats, showing the degree of fibrosis at each respective age. WT nude rats are shown on the left, and DMD nude rats are shown on the right. Scale bar = 1 mm (one-month-old), scale bar = 500 µm (5- and 12- month-old) **(B)** Detailed analysis of severe and progressive changes in the heart of a DMD nude rat. The upper row shows hematoxylin & eosin (H&E) staining, and the lower row shows SR staining images. The right panel shows magnified views of the left panel. Magnified area denoted by the square in panel **(A,B)** ×4, scale bar = 500 μm; ×10, scale bar = 50 µm. **(C)** Bar graph summarizing SR-positive areas in WT and DMD nude rats at 1, 5, and over 12 months of age. N.S. non-significant, *p < 0.05, **p < 0.01.

We performed histopathological evaluation of the heart using H&E and SR staining in 12-month-old DMD and WT nude rats due to the observed cardiac hypertrophy in DMD nude rats (Figure 2A). Figures 3A, B show histological images of the hearts from DMD and WT nude rats with H&E and SR staining. We observed minor fibrosis in the heart of 1-month-old DMD nude rats compared to that in WT nude rats. However, the fibrotic area expanded progressively in the hearts of 5 and 12-month-old DMD nude rats (Figure 3A), indicating progressive fibrosis of the heart with age in DMD nude rats. In addition to severe fibrosis, mononuclear cells and necrotic myofibers were also observed in the outer layer of the left and right ventricular walls of the hearts in DMD nude rats (Figure 3B). We evaluated the progression of fibrosis by quantifying the percentage of fibrotic area in transverse sections of the hearts using SR staining and observed an age dependent increase in the fibrotic area in DMD nude rats compared to that in WT nude rats (Figure 3C).

### 3.4 Pathological analysis of the diaphragm in DMD nude rats

**FIGURE 4:**
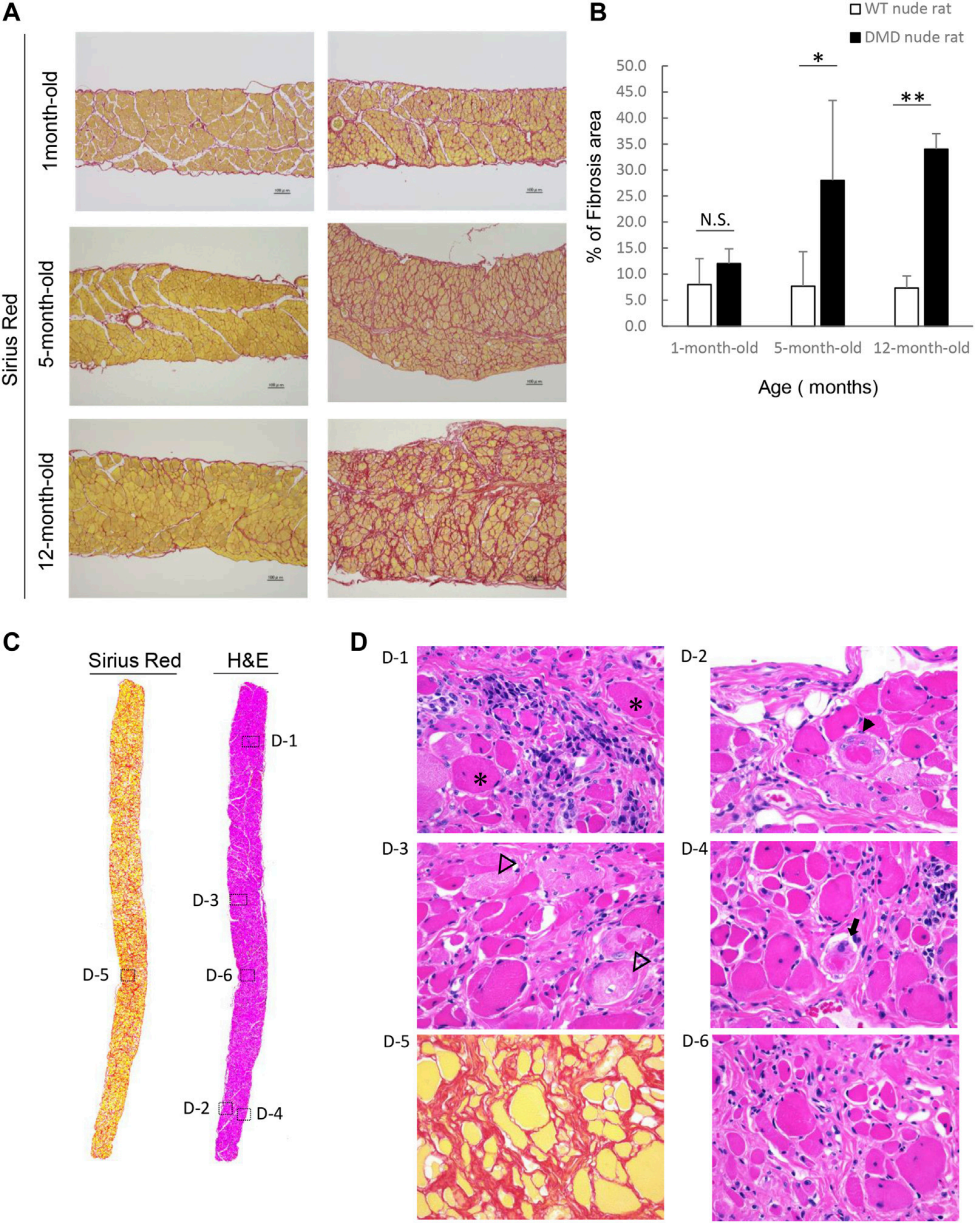
Detailed analysis of severe changes in the diaphragm in DMD nude rats. **(A)** Representative Sirius red (SR)-stained images of the detailed analysis of severe and progressive changes in the diaphragm in DMD nude rats. The left row shows the results from wild type (WT) nude rats, and the right row shows the results from DMD nude rats. Scale bar = 100 µm. **(B)** Bar graph summarizing SR-positive areas in the diaphragm from rats at 1, 5, and over 12-month-old. N.S. non-significant, **p < 0.01. **(C)** Representative images of SR and hematoxylin & eosin (H&E) staining showing the degree of fibrosis in the diaphragm of a DMD nude rat. The right row shows H&E staining, and the left row shows SR staining. **(D)** Representative images of H&E staining of the diaphragm of 12-month-old DMD nude rats. Magnified area denoted by the square in panel **(C)**. The symbols, *, ▼, ▽, and ➡, indicate hypercontraction (opaque) fiber (D-1), ring-fiber (D-2), pale necrotic fiber (D-3), and moth-eaten fiber (D-4), respectively. Comparative images of H&E and SR staining of the same location are shown (D-5 & D-6).

**FIGURE 5:**
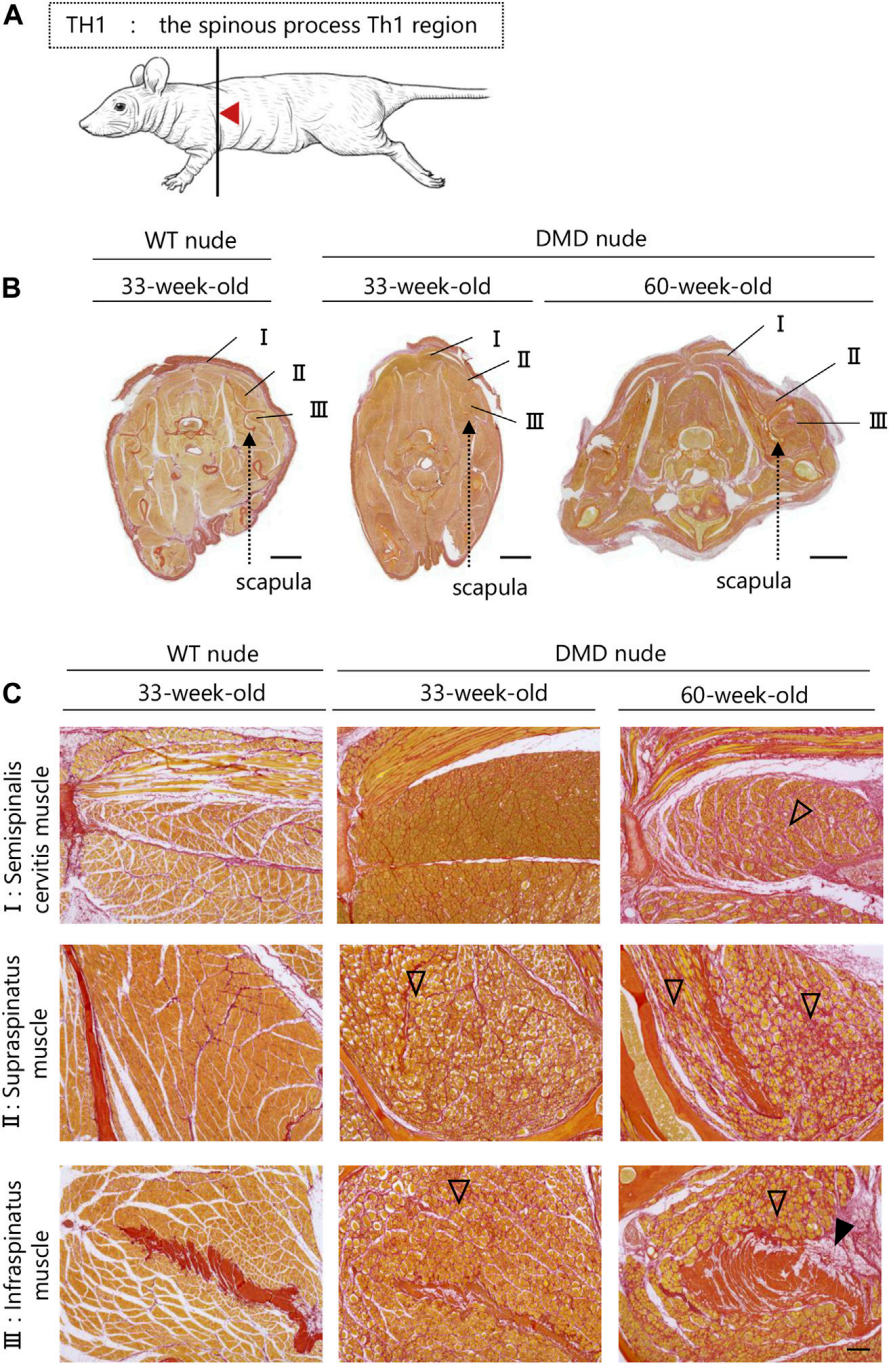
Lesion distribution within the skeletal muscle in transverse sections in DMD nude rats. **(A)** Schematic drawing showing the sectioning location of the shoulder and rump. The cuts were made in the spinous process of the Th1 region (Th1). **(B)** Representative images of Sirius red (SR) staining in Th1 shoulder sections of each rat type to evaluate the degree of fibrosis. Scale bar = 5 mm. **(C)** Magnified views of I, II, and III indicated by arrows are shown in the lower columns. Scale bar = 300 µm. The symbols, ▽ and ▼, indicate fibrosis and adipogenesis, respectively.

**FIGURE 6:**
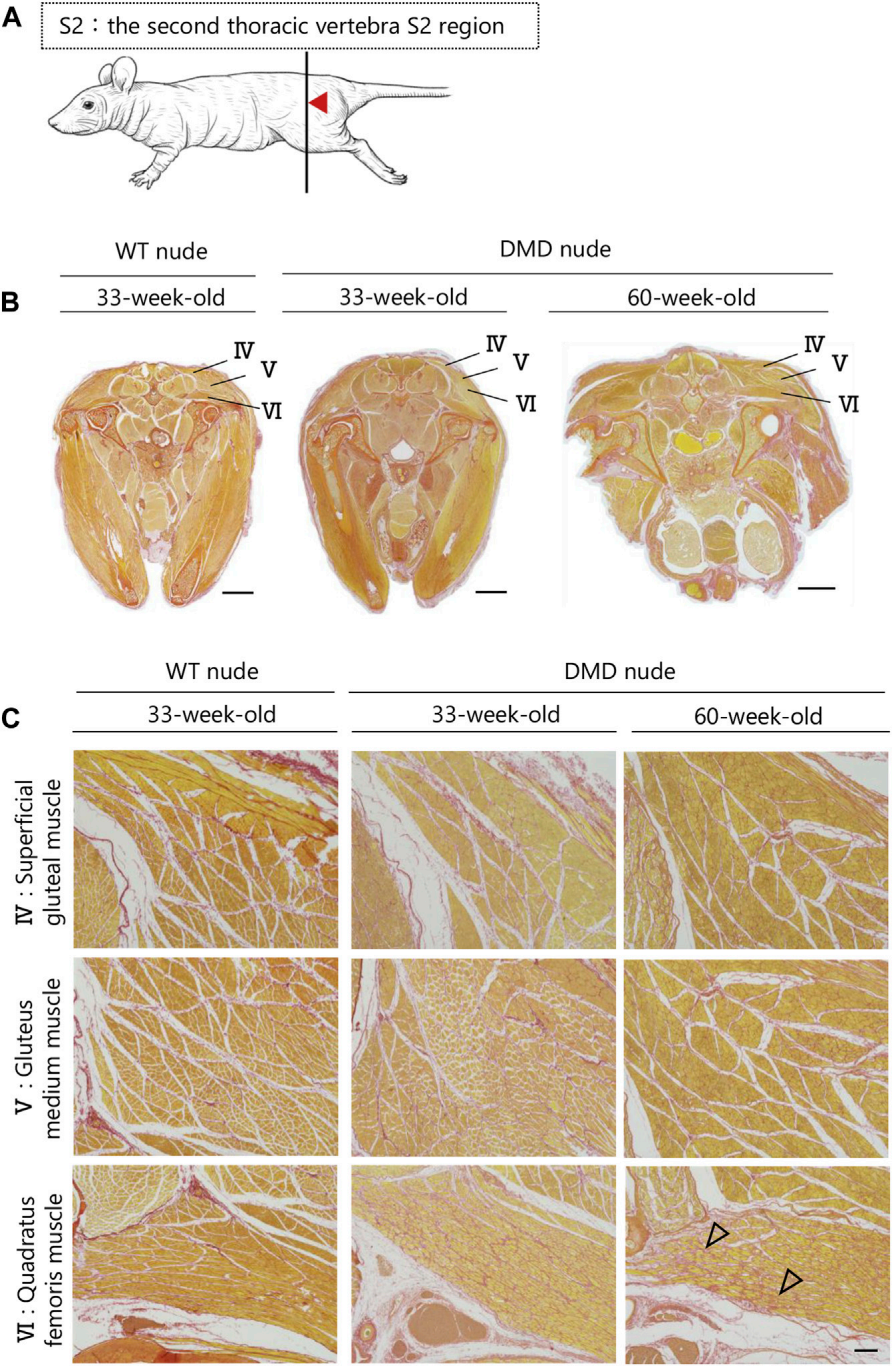
Lesion distribution within the skeletal muscle in transverse sections in DMD nude rats. **(A)** Schematic drawing showing the sectioning location of the rump. The cuts were made in the spinous process of the second thoracic vertebra S2 region (S2). **(B)** Representative images of Sirius red (SR) staining at the S2 lumbar section of each type of rat to evaluate the degree of fibrosis. Scale bar = 5 mm. **(C)** Magnified views of IV, V, and VI from **(B)** indicated by arrows are shown in the lower columns. Scale bar = 300 µm. The symbols, ▽ indicates fibrosis.

We examined fibrosis of the diaphragm using H&E and SR staining as decreased respiratory function is a characteristic of DMD. Figure 4A shows the histological images of the diaphragms from DMD and WT nude rats with SR staining. 1-month-old DMD nude rats displayed mild fibrosis in the diaphragm compared to that in WT nude rats. We observed progressing fibrosis in the diaphragm in 5- and 12-month-old DMD nude rats. We evaluated the progression of fibrosis by quantifying the percentage of fibrotic area using SR staining. Similar to that in the heart, we observed an age dependent increase in the fibrotic area in DMD nude rats compared to that in WT nude rats (Figure 4B). Figure 4C shows H&E- and SR-stained cross-sections of the diaphragms of 12-month-old DMD nude rats. The H&E-stained image shows a marked difference in the size of muscle fiber, while the SR-stained images show fibrotic areas throughout the diaphragm (Figure 4C). Magnified the H&E-stained images show Opaque fiber (Figure 4 D–1), ring-fiber (Figure 4 D–2), pale necrotic fiber (Figure 4 D–3), and moth-eaten (Figure 4 D–4). Progressive fibrosis in the interstitium (Figure 4 D–5), regenerating muscle with small muscle fiber caliber and central nuclei observed (Figure 4 D–6). These characteristic pathological findings in the diaphragms of DMD nude rats are typical human DMD patient-like diaphragmatic lesions.

### 3.5 Transverse histological analysis of whole muscle tissue of DMD model rats

A transverse sectional analysis was performed to comprehensively evaluate the body’s musculature. The muscles in the shoulder region were analyzed by sectioning the spine at the Th1 position (Figure 5A). Figures 5B, C and Supplementary Figure S2 present the representative H&E and SR-stained images of the shoulder and buttock muscles from 33 to 60-week-old DMD and WT nude rats. The skeletal muscles around the scapula of the Th1 region showed increased fibrosis in 60-week-old DMD nude rats (Figure 5B), and progressive fibrosis was also observed in semispinalis cervitis (I), supraspinatus muscle (II), and infraspinatus muscle (III) with positive SR staining (Figure 5C). The muscles in the buttock region were analyzed by sectioning the lumbar vertebra at the S2 position (Figure 6A). We observed similar levels of fibrosis between DMD and WT nude rats in the gluteal muscles, including the superficial gluteal (IV) and gluteus medium (V) muscles. We did not observe pronounced fattening (Figures 6B, C; Supplementary Figure S2), in contradiction to that reported in the magnetic resonance imaging results of human patients with DMD (Polavarapu et al., 2016). However, the muscles associated with moving joints, such as the quadratus femoris muscle (VI), were revealed to be impaired (Figure 6C).

### 3.6 Validation of human cell engraftment in DMD nude rats

**FIGURE 7:**
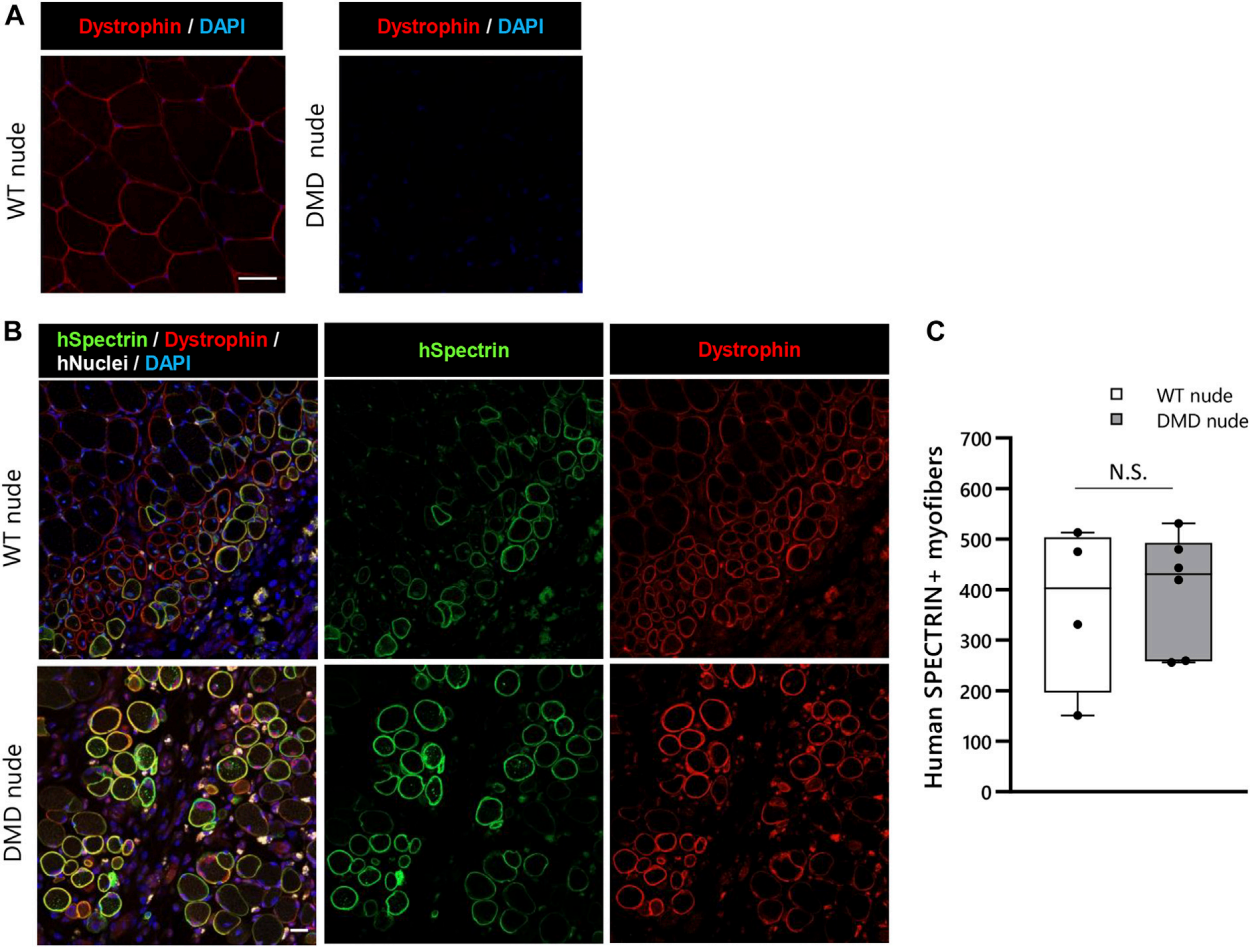
Suitability of the immunodeficient DMD rat model for engraftment after human cell transplantation. **(A)** Representative images of immunostaining with dystrophin antibodies in cryosections of the TA muscle of WT nude rat. **(B)** Representative images showing engraftment of transplanted Hu5/KD3 cells in 4-week-old WT and DMD nude rats. The engrafted cells were evaluated 3 weeks after transplantation using immunohistochemistry for human spectrin. Human spectrin (green), dystrophin (red), nuclei (white), and DAPI (blue). Scale bars = 25 µm. **(C)** The number of engrafted myofibers in 1 TA muscle. N.S. non-significant.

We conducted transplantation studies to evaluate the acceptance of human cells by WT and DMD nude rats. First, we performed immunohistochemistry using an anti-dystrophin antibody to confirm the absence of dystrophin protein expression in DMD nude rats (Figure 7A). After cryo-injury treatment, we injected immortalized human myoblasts (Hu5/KD3) into the TA muscles of WT and DMD nude rats. Three weeks post-transplantation, the rats were euthanized, and TA muscles were collected for cryosectioning. Evaluation of human myoblast engraftment, through the detection of human spectrin-positive fibers (Figure 7B), revealed almost 400 human spectrin-positive myofibers in both DMD and WT nude rats (Figure 7C). We observed dystrophin expression in the spectrin-positive fibers in the DMD nude rat muscle (Figure 7B), indicating that regeneration of dystrophin positive myofibers by human cells can be assessed using the DMD nude rat. In WT nude rats, Hu5/KD3 cells were still engrafted 3 months after transplantation (Supplementary Figure S3). On the other hand, when Hu5/KD3 cells were transplanted into the immunocompetent DMD model rats (X^Dmd^Y Foxn1^+/rnu^), no engraftment was observed (Supplementary Figure S4).

## 4 Discussion

In this study, we generated an immunodeficient DMD rat model (DMD nude rat) and confirmed that it exhibited the DMD pathology similar to that seen in human patients. Furthermore, we confirmed that engraftment of immortalized human myoblasts followed by muscle regeneration could be assessed in DMD nude rats.

Several DMD model animals have been established and reported to date, including dogs (Kornegay, 2017), rats (Larcher et al., 2014; Nakamura et al., 2014; Taglietti et al., 2022), and mice (Tanabe, 1986). However, to study the use of cell transplantation as a possible therapy for DMD, immunodeficient animal models should be used to enable grafting different types of donor cells without the risk of immune rejection. The NSG-mdx^4Cv^ mouse has been reported as an optimal immunodeficient DMD model enabling the engraftment of human cells (Arpke et al., 2013). The mdx4^Cv^ mouse harbors a point mutation in exon 53 of the *dmd* gene resulting in a premature stop codon (Im et al., 1996) and has been used for validating dystrophin restoration procedures because it has 10-times fewer revertant fibers than the C57BL/10-mdx mouse (Danko et al., 1992). The NSG-mdx^4Cv^ mouse was established by breeding the mdx^4Cv^ mouse with the NSG mouse, which is severely immunodeficient, lacking T, B, and NK cells (Cao et al., 1995) and shows significant engraftment of transplanted xenogeneic cells (Arpke et al., 2013). Although the NSG-mdx^4Cv^ mouse displayed slightly enhanced fibrosis in the diaphragm and quadriceps compared to that in the C57BL/10-mdx mouse, no adipogenesis was observed in the NSG-mdx^4Cv^ mouse. Moreover, muscle function was identical between the NSG-mdx^4Cv^ and C57BL/10-mdx mice, revealing a milder DMD phenotype than that exhibited in human patients (Arpke et al., 2013). DMD nude rat model is characterized by lower body weight and shorter life span compared to WT nude rats. DMD nude rats had significantly lower body weight than WT nude rats at 4 weeks of age and died by 68-week-old. In contrast, C57BL/10-mdx mice show no significant weight loss compared to controls (Tanabe, 1986) and have a mean lifespan that is 19% reduce than controls at 21.5-month-old (Chamberlain et al., 2007). Furthermore, the forelimb grip strength of C57BL/10-mdx mice show 13.0% of reduction from WT mice (Spurney et al., 2009), while DMD nude rats show 36.0% reduction from WT mice at 5-6-month-old.We also observed increased muscle breakdown markers including serum CK activity and urine titin level, in the early stages of disease progression, which decreased as the disease progressed. Thus, our DMD nude rat model showed a more severe phenotype than the C57BL/10-mdx mouse, making it suitable for evaluating the efficacy of cell transplantation therapy in preclinical studies.

Next, we performed histopathological evaluation of the degree of skeletal muscle degradation and observed overall decreased skeletal muscle weights in DMD nude rats compared to those in WT nude rats. In addition, we observed progressive fibrosis of the heart and diaphragm, which are closely related to rat survival, in DMD nude rats, with increasing severity as the rats aged. SR-stained images also showed advanced fibrosis and adipogenesis, with markedly small fiber diameter in the quadriceps muscle of DMD nude rats, which has not been reported in the DMD mouse model. DMD nude rats showed severe fibrosis and adipogenesis of skeletal muscle, similar to previously reported DMD rat models (Larcher et al., 2014; Nakamura et al., 2014; Taglietti et al., 2022). In DMD nude rat, muscle fibers of different sizes due to repeated necrosis and regeneration of muscle fibers characteristic of patient with DMD were observed. Cardiac fibrosis and respiratory fibrosis also progressively and age-dependently worsen, comparable to previously reported DMD rat models (Larcher et al., 2014; Nakamura et al., 2014; Taglietti et al., 2022). A transverse histological analysis of DMD nude rat muscle tissues revealed damaged muscles around the scapula in the shoulder area. In contrast, despite progressive fibrosis of the deep gluteal muscles, we did not observe any severe damage to the buttocks. The lack of adipogenesis in the buttocks, unlike that seen in human patients with DMD (Polavarapu et al., 2016), could be explained by differences between bipedal and tetrapodal animals (Vlahovic et al., 2017; Veenvliet et al., 2020). Further, we confirmed that our DMD nude rats can receive transplanted human myoblasts for preclinical studies. The injected Hu5/KD3 cells showed similar engraftment in WT and DMD nude rats, analyzed using spectrin and dystrophin positive human fibers. Moreover, we detected Hu5/KD3 cells in the TA muscles in WT nude rats *via* positive spectrin and dystrophin staining even after 3 months, indicating strong engraftment. Notably, since DMD nude rats show no revertant fiber which some DMD model rats have (Larcher et al., 2014; Taglietti et al., 2022), such phenotype would be an advantage to assess the engraftment efficiency accurately after cell transplantation. Future cell transplantation studies should be conducted using these DMD nude rats to administer skeletal MuSCs derived from iPS cells (Zhao et al., 2020) and to evaluate their engraftment and function. However, we observed some instances of accidental death with isoflurane anesthesia at about 5 months of age and after treadmill exercise at the age of 7 months (data not shown). Additionally, since natural breeding of DMD nude rat is difficult, we have confirmed that artificial insemination can produce fertilized eggs, although the probability is low (data not shown). Thus, studies should be carefully designed to analyze the effects of cell therapy and other drugs, because the symptoms of DMD nude rat are more severe than those of NSG-mdx^4Cv^ mouse.

In summary, we have developed immunodeficient DMD rat model that exhibits a pathology similar to human patients with DMD and that accepts human myoblast transplantation, making it suitable for basic DMD research as well as for preclinical studies investigating cell transplantation therapy. We report no revertant fiber and an appropriately sized animal model for evaluating skeletal muscle after human MuSC transplantation with more severe symptoms than in the previously established NSG-mdx^4Cv^ mouse model with milder symptoms.

## Data Availability

The original contributions presented in the study are included in the article/Supplementary Material, further inquiries can be directed to the corresponding author.
