# Supplementary material for: A new immunodeficient Duchenne muscular dystrophy rat model to evaluate engraftment after human cell transplantation
Source: Front Physiol. 2023 Apr 10;14:1094359. doi: 10.3389/fphys.2023.1094359 (PMC10123282; doi:10.3389/fphys.2023.1094359)
Supplement: Supplementary file 1 [file DataSheet1.pdf]

Supplementary Table S1

1st antibody	Sourse	Clonarity	Dilution	Company
Anti-Dystrophin antibody	rabbit poly	IgG	1:100 or 1:200	Abcam
Anti-h-Spectrin antibody	mouse mono	IgG2b	1:100	Leica
Anti-h-Nuclei antibody	mouse mono	IgG1	1:500	Merck
Anti-Lamin A/C antibody	mouse mono	IgG2b	1:200	Santacruts
Anti-Laminin α2 antibody	rat mono	IgG1	1:50	ALEXIS
**2nd antibody**	**Sourse**	**Clonarity**	**Dilution**	**Company**
Anti-Rabbit antibody 488			1:500	Thermo Fisher Scientific
Anti-Rabbit antibody 568			1:500	Thermo Fisher Scientific
Anti-mouse IgG1 antibody 568			1:500	Thermo Fisher Scientific
Anti-mouse IgG1 antibody 647			1:500	Thermo Fisher Scientific
Anti-mouse IgG2b antibody 488			1:500	Thermo Fisher Scientific
Anti-mouse IgG2b antibody 568			1:500	Thermo Fisher Scientific
Anti-Rat antibody 647			1:500	Abcam
Anti-mouse IgG1 antibody HRP			1:500	Abcam

Supplemental Table S1. List of antibodies used in the study and their respective dilutions.

Supplementary Figure S1

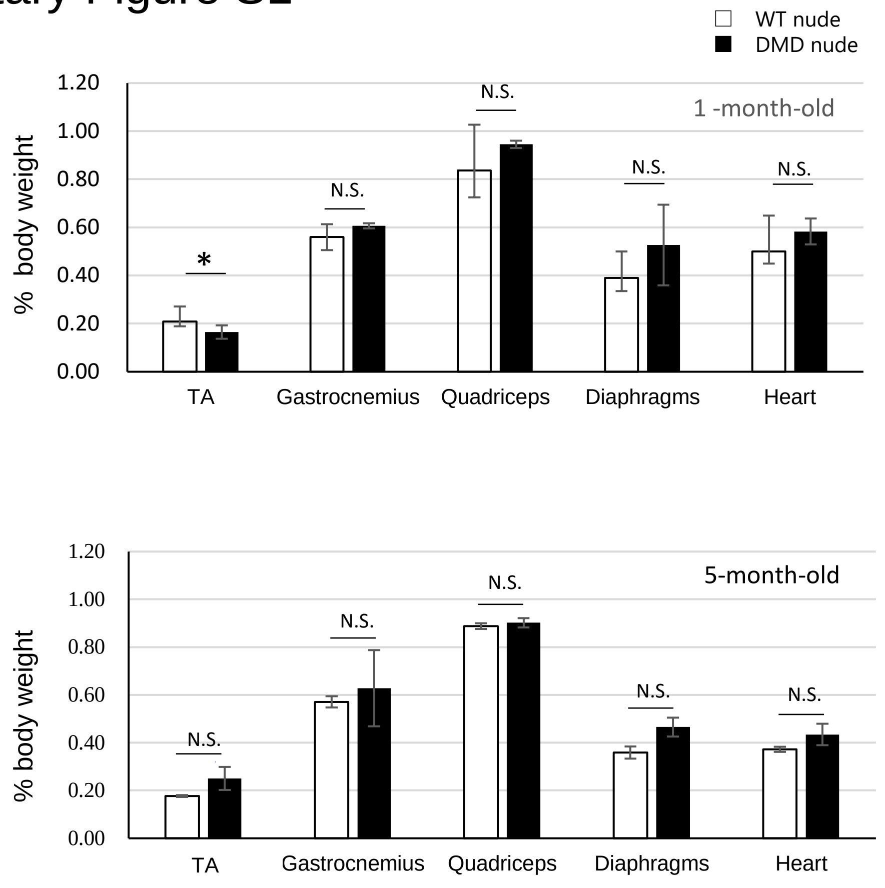

Supplementary Figure S1. Analysis of skeletal muscle to body weight ratio in Duchenne Muscular Dystrophy (DMD) and wild type (WT) nude rats at 1 and 5-month-old.

Supplementary Figure S2

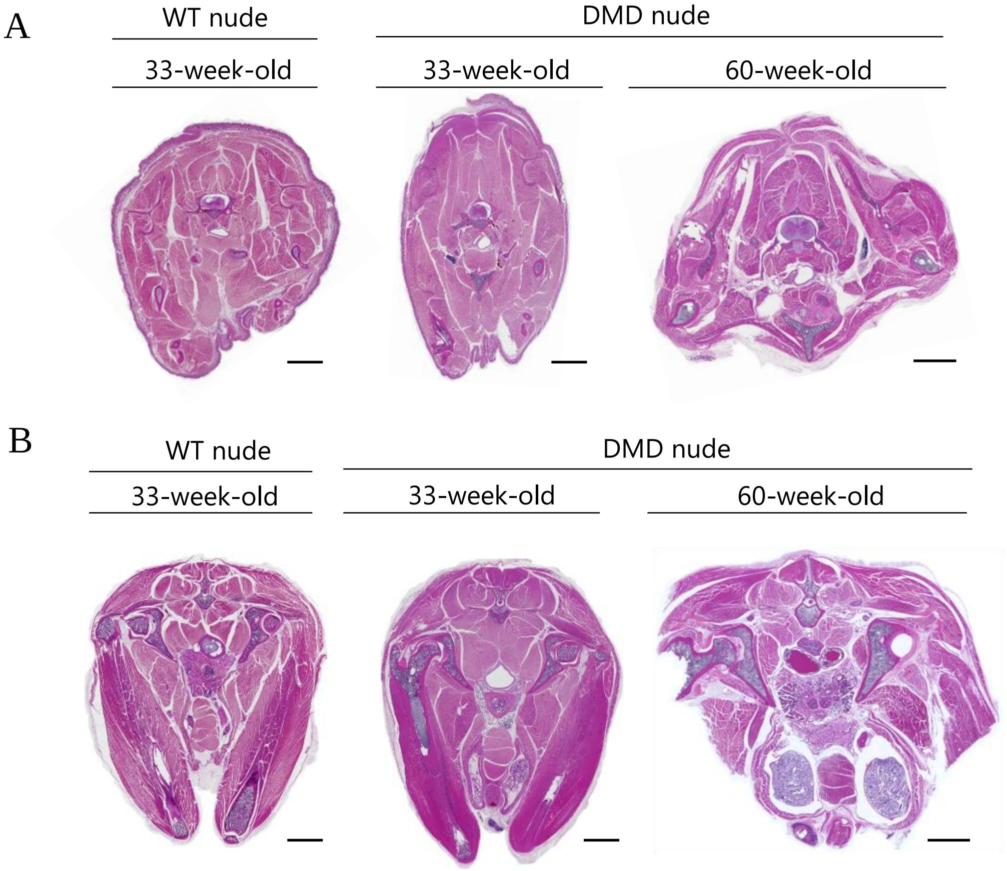

Supplementary Figure S2. Hematoxylin & eosin (H&E) staining images in the Th1 and S2 region of wild type (WT) nude rats and Duchenne Muscular Dystrophy (DMD) nude rat. A shows an overall view of a section cut at position Th1. Scale bar = 5mm. B shows an overall view of a section cut at position S2. Scale bar =300μm.

Supplementary Figure S3

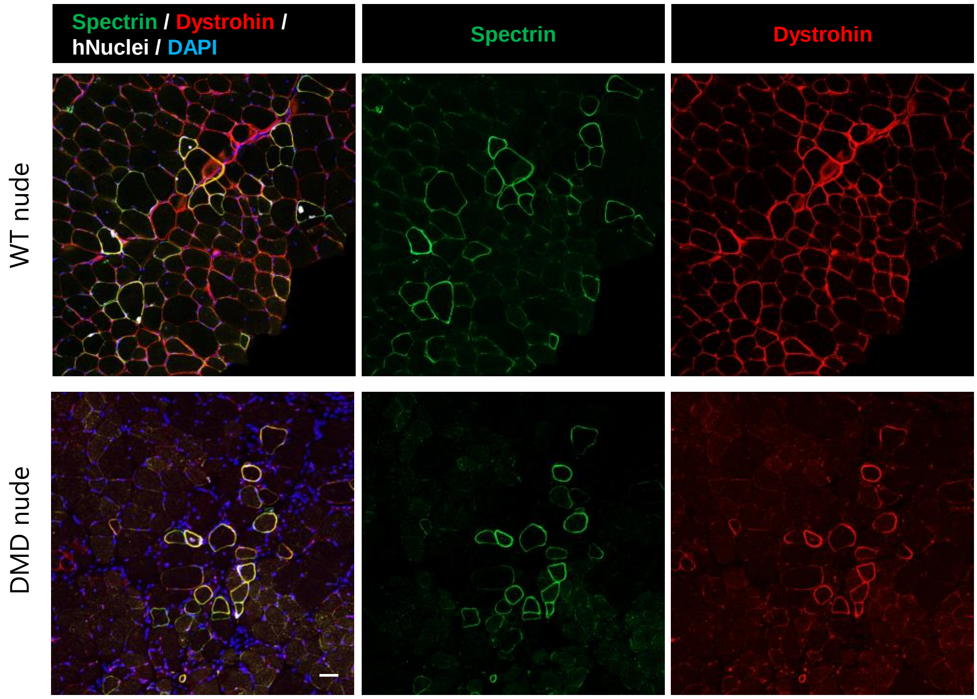

Supplementary Figure S3. Confirmation of long-term (3 months) engraftment of human cells in wild type (WT) nude rats and Duchenne Muscular Dystrophy (DMD) nude rat. Scale bar = 25 µm.

Supplementary Figure S4

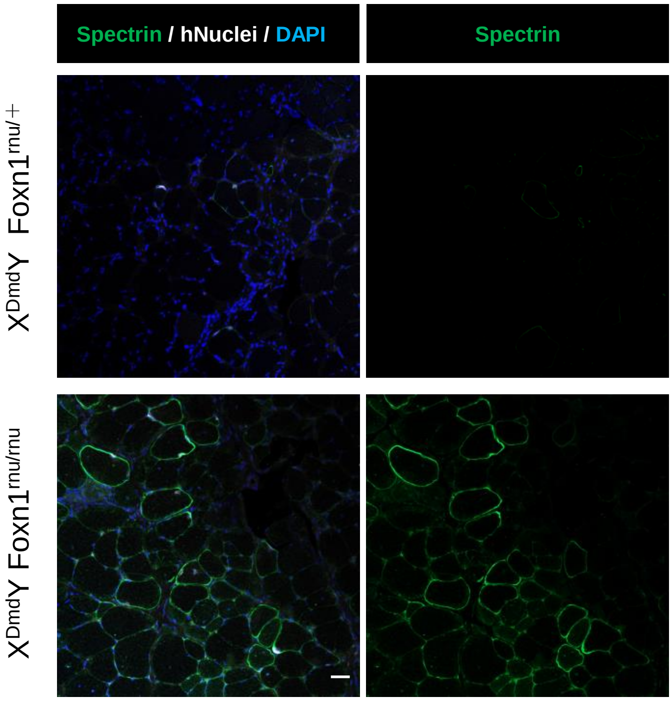

Supplementary Figure S4. Comparison of human cell engraftment between immunocompetent ($Foxn1^{rnu/+}$) and immunodeficient ($Foxn1^{rnu/rnu}$) DMD model rats. Scale bar = 25 µm.
